# Elevated sortilin expression discriminates functional from non-functional neuroendocrine tumors and enables therapeutic targeting

**DOI:** 10.3389/fendo.2024.1331231

**Published:** 2024-04-17

**Authors:** Felix Bolduan, Alexandra Wetzel, Yvonne Giesecke, Ines Eichhorn, Natalia Alenina, Michael Bader, Thomas E. Willnow, Bertram Wiedenmann, Michael Sigal

**Affiliations:** ^1^Department of Hepatology & Gastroenterology, Charité Universitätsmedizin Berlin, Berlin, Germany; ^2^Berlin Institute of Health at Charité – Universitätsmedizin Berlin, BIH Biomedical Innovation Academy, BIH Charité Junior Digital Clinician Scientist Program, Berlin, Germany; ^3^Max-Delbrück-Center for Molecular Medicine in the Helmholtz Association (MDC), Berlin, Germany; ^4^German Center for Cardiovascular Research (DZHK), Berlin, Germany; ^5^Charité - Universitätsmedizin Berlin, Berlin, Germany; ^6^University of Lübeck, Institute for Biology, Lübeck, Germany; ^7^Department of Biomedicine, Aarhus University, Aarhus, Denmark; ^8^Berlin Institute for Medical Systems Biology (BIMSB), Max Delbrück Center for Molecular Medicine in the Helmholtz Association (MDC), Berlin, Germany

**Keywords:** neuroendocrine tumors, functional syndrome, carcinoid syndrome, serotonin, sortilin, organoids, enteroendocrine cells

## Abstract

A subset of neuroendocrine tumors (NETs) can cause an excessive secretion of hormones, neuropeptides, and biogenic amines into the bloodstream. These so-called functional NETs evoke a hormone-related disease and lead to several different syndromes, depending on the factors released. One of the most common functional syndromes, carcinoid syndrome, is characterized mainly by over-secretion of serotonin. However, what distinguishes functional from non-functional tumors on a molecular level remains unknown. Here, we demonstrate that the expression of sortilin, a widely expressed transmembrane receptor involved in intracellular protein sorting, is significantly increased in functional compared to non-functional NETs and thus can be used as a biomarker for functional NETs. Furthermore, using a cell line model of functional NETs, as well as organoids, we demonstrate that inhibition of sortilin reduces cellular serotonin concentrations and may therefore serve as a novel therapeutic target to treat patients with carcinoid syndrome.

## Introduction

Neuroendocrine tumors (NETs) are rare malignant neoplasms that can occur as primary tumors in every organ but are most frequently found in the gastroenteropancreatic (GEP) system. NETs consist of cells displaying neuronal and endocrine characteristics (1, 2). Since 1975, the age-adjusted incidence rate of GEP-NETs in the US has increased by 6.4 times to 5.45 per 100,000 persons (3). In approximately one-third of cases, NETs secrete excessive amounts of peptidic hormones and biogenic amines into the circulation, resulting in a hormone-mediated disease. These tumors are called “functional”. One of the most common hormone-mediated disease is carcinoid syndrome, caused primarily by over-secretion of serotonin, which occurs in 32% of all small intestinal NETs (4). Metastatic grade small intestinal NET patients suffering from this syndrome have a significantly shorter median overall survival (4.7 years, Confidence interval 4.0-5.4) than those without functional syndrome (7.1 years, CI 5.2-8.1) (4, 5). It is widely accepted that in order to cause carcinoid syndrome, cells need to evade the first-pass effect of the liver (e.g., due to liver metastases, widespread retroperitoneal metastases, ovarian metastases, or bronchial primaries). However, morphologically it is not possible to discriminate functional from non-functional tumors and comparative molecular analyses have so far failed to reveal obvious cellular markers of functional tumors. Identification of specific features of functional cells may not only provide insights into the tumor biology of NETs but also reveal novel and highly needed therapeutic targets.

Sortilin, also known as neurotensin receptor 3, is a member of the VPS10P domain receptor family - a group of transmembrane receptors involved in uptake as well as intracellular sorting of a broad range of protein ligands (6). Recent studies focusing on the role of sortilin in cancer uncovered that this receptor is expressed in many cancer cells, including breast and lung cancer. Its expression has been associated with a variety of effects, ranging from pro-tumorigenic to tumor-suppressive (7–9). Expression of sortilin was also detected in NETs and linked to cell migration and adhesion (10). Interestingly, only a fraction of tested NET samples was positive for sortilin expression, raising the question of what distinguishes sortilin-positive and sortilin-negative tumors. As sortilin plays an important role in regulating secretion of neurotrophins in neurons (11, 12), we hypothesized that this receptor may be essential for the ability of NETs to produce and secrete hormones and thereby serves as a key factor in the development of functional syndrome.

In this study we aim to demonstrate that elevated sortilin expression is a novel feature of hormonally active NETs. By comparative sortilin-immunostaining we could indeed confirm this hypothesis. Furthermore, we demonstrated that sortilin inhibition causes reduced levels of serotonin in cell culture systems. Thereby, sortilin serves as a novel potential target in treatment of the functional syndrome of NETs.

## Methods

### Neuroendocrine tumor tissue

Paraffin-embedded tissue of neuroendocrine tumor samples was used for this study. The collection of tissue samples for this study was approved by the Ethics Committee of the Charité - Universitätsmedizin Berlin (No EA1/229/17). 1 mm punches with a thickness of 2 µm were obtained and plated on tissue microarrays (TMA). For each sample, 3 replicates were taken. Tissue was rehydrated, antigen retrieval was performed using 10 mM citrate buffer and incubated for 20 min in Avidin/Biotin Blocking Solution (Dako, X0590). Slides were washed in phosphate-buffered saline (PBS, Gibco) and incubated for 10 min in 0.1% Triton X-100 in PBS. After further washing steps, the tissue was incubated overnight at 4°C with anti-sortilin antibody (1:100, af3154, R&D systems). After repeated washing steps, tissue was incubated for 30 min at room temperature with a secondary biotin-linked anti-goat antibody (1:300, Dako, E0466), followed by incubation with ABC complex solution (Vector, PK-6100) for 30 min. After an additional washing step with PBS, a DAB buffer solution (Dako, 3468) was applied according to the manufacturer’s instruction until a color change occurred (approx. 10 min) before stopping the reaction with distilled H2O. Staining with hematoxylin was performed and tissue embedded in glycerin-gelatine.

In total, 49 tumor samples [21 neuroendocrine primary tumors (7 pancreatic NETs and 14 small intestinal NETs; of these 14 functional and 7 non-functional) and 28 liver metastases of neuroendocrine tumors (10 pancreatic, 1 lung, 15 small intestinal, and 2 NETs of unknown origin; of these, 16 functional and 12 non-functional)] were included in this study. To exclude the possibility of high hormonal secretion without symptoms in small intestinal NETs due to the first-pass effect of the liver, only primary tumors that caused hepatic metastases were included. Tissue was examined under brightfield microscopy and immunoreactivity scores were obtained by multiplication of the staining intensity (0, no staining; 1, weak staining; 2, moderate staining; 3, strong staining) and the percentage of positively stained cells (0, no positive cells; 1, 0%-10% positive; 2, 11%-50% positive; 3, 51%-100% positive). Each sample consisted of three replicates and the final score for each sample was obtained by averaging the immune reactivity scores of all three. Negative controls without primary antibody were established for every sample.

### Cell culture

BON cells, a kind gift from Courtney Townsend (University of Texas, Galveston, TX), were cultured in RPMI 1640 medium containing L-glutamine (Gibco) supplemented with 10% FBS at 37°C with 5% CO2 in O2. During the exponential growth phase, cells were treated with the sortilin inhibitor AF38469 (10 µM, MedChemExpress) for 24 h. In order to exclude toxic effects, cell viability measurements were performed. After 24 h incubation with 10 µM AF38469 or vehicle, cells were harvested and counted using Countess II™ (Invitrogen). They were further treated with trypan blue (Invitrogen) and alamarBlue™ (Thermo Fisher Scientific) to determine the percentage of live cells and cell proliferation, respectively.

### Western blot assay

NET tissue or BON cells were washed with PBS (Gibco), lysed with RIPA buffer (Thermo Fischer Scientific) containing a protease inhibitor cocktail (cOmplete™ Mini, Roche) and sonication (10s, 60% intensity). The proteins were separated by SDS-PAGE (10-12% Tris-Glycin, WedgeWell™, Invitrogen) and transferred onto PVDF membranes (Bio-Rad). After blocking with 5% nonfat dry milk, the membranes were incubated with primary antibodies against sortilin (1:250, AK BD#612101, Becton Dickenson) and α-tubulin (1:1000, #T9026, Sigma) overnight at 4°C, followed by incubation with secondary anti-mouse IgG antibody (1:10000, #AB_2340061, Jackson Immuno Research) for 1 h at room temperature. Detection was performed with SuperSignal™ West Dura Extended Duration Substrate (Thermo Fisher Scientific) using the Molecular Imager^®^ VersaDoc™ and quantified with Image Lab™ software (Bio-Rad).

### Primary mouse small intestinal organoid culture

Experiments and animal maintenance were performed in accordance with local (LaGeSo, Berlin, T-CH0032/20), national (German Animal Welfare Act), and international guidelines (EU Directive 2010/63/EU). Male 6 to 12-week-old C57BL/6 mice obtained from Charles River Laboratory were used for this study. For the generation of murine small intestinal organoids, animals were sacrificed by cervical dislocation, the ileum was dissected, washed with ice-cold PBS (Gibco), and opened longitudinally. The villi were gently removed using a glass coverslip. 5 mm-long pieces were washed 10 times in ice-cold PBS, followed by incubation for 5 min in 10 mM EDTA (Invitrogen)/PBS at room temperature and 30 min in 2 mM EDTA/PBS supplemented with 2.5 µM DTT (Sigma) at 4°C on a rotating shaker. After removing the supernatant, the pieces were shaken vigorously with 5 ml HBSS containing magnesium and calcium chloride (Gibco) and 10 ml PBS. The supernatant was filtered through a 70 µm filter (Greiner Bio-One) and centrifuged at 200 x g for 3 min at 4°C. The pellet was resuspended with 3 ml 0.1% BSA/PBS and the number of crypts determined. After a repeated centrifugation step, 2,000 crypts per 10 µl Cultrex Basement Membrane Extract Type 2 (R&D systems) were seeded in 48-well plates. The organoids were cultured in Advanced Dulbecco’s Modified Eagle’s Medium/F12 (Gibco) consisting of 10 mM HEPES (Gibco), 2 mM GlutaMAX (Gibco), and 10% penicillin/streptomycin (Gibco), supplemented with 1.25 mM N-acetylcysteine (Sigma), 25% R-spondin 1 conditioned medium, 1× B-27 (Gibco), 1× N2 (Gibco), 50 ng/ml mEGF (Invitrogen), and 100 ng/ml mNoggin (PeproTech). The medium was replaced every 2-3 d and the organoids were passaged mechanically after 5-7 d using a syringe and needle.

### EEC differentiation

Three days after passaging, ileal organoids were treated with the above-described medium but without mEGF and supplemented with a Notch inhibitor (10 µM DAPT, Sigma) and a MEK1 and 2 inhibitor (1 µM PD0325901, Sigma) for four days. To confirm the enrichment of EECs, expression of the marker synaptophysin was determined using immunofluorescence staining. Organoids were harvested, washed with PBS, and fixed with 3.7% paraformaldehyde overnight at 4°C, followed by incubation in 0.1% BSA/PBS for at least 30 minutes. After embedding in 2% agarose, organoids were dehydrated, embedded in paraffin, and cut into 4 µm sections. Sections were deparaffinized and rehydrated, followed by antigen retrieval in 10 mM citrate buffer (pH 6). To avoid unspecific antibody binding, sections were incubated with blocking buffer (0.1% Tween/PBS supplemented with 5% FBS) for 2 h at room temperature, followed by incubation with the primary antibody against synaptophysin (1:100, ab178412, clone EPR1097-2, Abcam) overnight at 4°C. Sections were washed three times with 0.1% Tween/PBS, incubated with the secondary antibody AlexaFluor 647 donkey anti-rabbit IgG (1:400, Jackson Immunoresearch, 711-606-152) and DAPI for 2 h at room temperature, mounted by using an aqueous mounting medium and covered with a glass cover slip.

### Immunofluorescence staining of serotonin

For immunofluorescence staining of serotonin after sortilin inhibition, 2,000 BON cells were trypsinized, seeded onto glass slides, and fixed with methanol/acetone (1:1) for 2 min at room temperature. After washing with PBS and blocking with 5% goat serum in PBS (Biochrom) for 30 min at room temperature, the cells were incubated with the primary antibody against serotonin (1:400, #M0758, clone 5HT-H209, Dako) for 1 h at room temperature. The cells were washed three times for 2 min with PBS and incubated with Cy3-Goat anti-mouse IgG (1:1000, Jackson Immuno Research, 111-225-144) for 30 min at room temperature. After repeated washing with PBS, the cells were incubated with ethanol for 2 min and mounted in Immu-Mount™ (#9990402, Epredia).

All immunofluorescence images were acquired with the confocal laser scanning microscope TCS SP8 (Leica) or Observer 7 microscope (Zeiss). Images were collected and analyzed with Leica Application Suite X 3.5.6.21594 (LAS X, Leica) and ZEN 3.4 (Zeiss). Five images were collected per sample.

### Serotonin quantification

For quantification of serotonin concentrations, BON cells were treated with sortilin inhibitor AF38469 (10µM, MedChemExpress) or vehicle for 24 h and cultured in serum-free DMEM/HamsF12 (Biochrom), supplemented with 0.1% BSA and 1% penicillin/streptomycin. After 24 h, the supernatant (200 µl) was removed, precipitated with the HPLC lysis buffer containing 1.68% perchloric acid and 0.1 mg/ml ascorbic acid (both from Sigma-Aldrich) in H2O, and stored at -80°C. The cell layer was harvested with 200 µl HPLC lysis buffer. After centrifugation (20,000g for 20 minutes at 4°C), the supernatants containing the cell lysates were stored at -80°C.

Murine ileal organoids were cultured and differentiated as described above and treated without or with sortilin inhibitor AF38469 (10 µM, MedChemExpress) for 96 h during EEC differentiation. For the last 24 h of the experiment, organoids were kept in serum-free DMEM/HamsF12 (Biochrom) instead of Advanced Dulbecco’s Modified Eagle’s Medium/F12 without HEPES and Glutamax. Afterwards, the medium was removed and the organoids dissociated into single cells by incubating in TryplE (Gibco) for 5 min at 37°C followed by washing in PBS/BSA. After centrifugation, cells were counted and transferred to the HPLC lysis buffer. After centrifugation (14,000 g for 20 min at 4°C) the supernatants were stored at -80°C.

Serotonin measurements were performed using high-sensitive reversed-phase high-performance liquid chromatography (HPLC) with fluorometric detection as previously described (13). Briefly, separation of samples was performed over a C18 reversed phase column (LipoMare C18, AppliChrom, Oranienburg, and ProntoSIL 120 C18 SH, VDS Optilab, Berlin) in a 10 mM potassium phosphate buffer (pH 5.0) (Sigma-Aldrich, Steinheim, Germany) including 5% methanol (Roth, Karlsruhe, Germany) with a flow rate of 0.8-1.0 mL/min at 20°C. The excitation wavelength was 295 nm and the fluorescent signal was measured at 345 nm. Analyses of peak parameters of chromatographic spectra and quantification of substance levels, based on comparative calculations with alternately measured external standards, were performed by using the CLASS-VP software (Shimadzu, Tokyo, Japan).

### RNA isolation and quantitative reverse transcriptase PCR

Total RNA from BON cells or EEC-enriched ileal organoids was isolated with the NucleoSpin® RNA kit (Macherey-Nagel) according to the manufacturer’s instructions. RNA concentration and purity were determined with a NanoDrop™ OneC (Thermo Fisher Scientific). cDNA synthesis was performed using the iScript™ cDNA Synthesis Kit (Bio-Rad) and qRT-PCR performed using the PowerUp™ SYBR^®^ Green Master Mix (Thermo Fisher Scientific) on a QuantStudio™ 3 (Thermo Fisher Scientific). The qRT-PCR conditions were as follows: 42 cycles of 15s at 95°C, 15s at 58°C, and 30s at 72°C. Relative quantification was calculated with the ΔΔ Ct method with human hypoxanthine phosphoribosyltransferase 1 (HPRT1) and murin beta-microglobulin (ßMgi) as reference genes (**Table 1**).

**Table 1:** Primer sequences for gene expression analysis by qRT-PCR.

Target	fw	rv
h*TPH1*	TACTGGCGCCCGAG TTTTAG	GCACAATGGTCCAG GTCAGA
h*HPRT1*	CCCTGGCGTCGTGA TTAGTG	CGAGCAAGACGTTC AGTCCT
m*Syn*	TTGGCTTCGTGA AGGTGC	CTGCCGCACGTA GCAAAG
m*Chga*	AGAAGTGTTTGAGAAC CAGAGCCC	TTGGTGATTGGGTATTGG TGGCTG
m*ßMgi*	TTCTGGTGCTTGTCTC ACTGA	CAGTATGTTCGGCTTC CCATTC

h, human; m, murine.

### Statistical analysis

**Figure 1:**
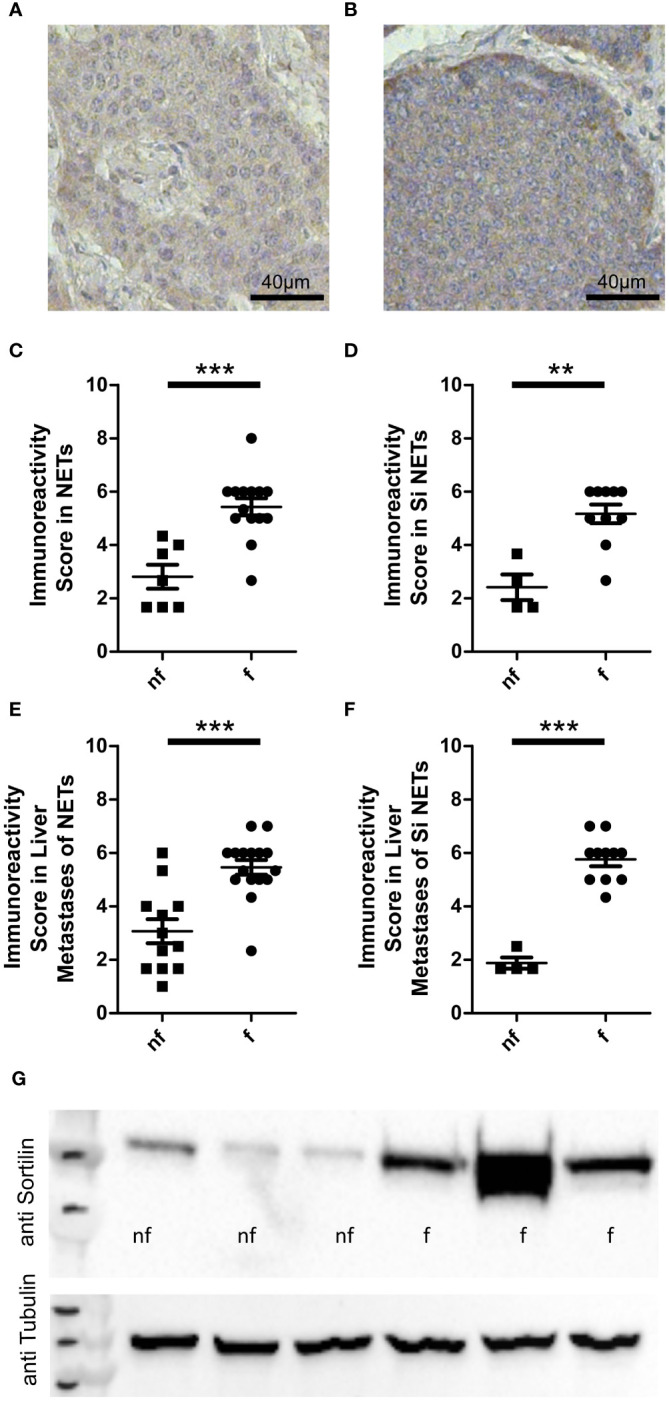
Sortilin expression in functional and non-functional neuroendocrine tumors (NETs). Neuroendocrine primary tumor tissue sections (n=21) and liver metastases of neuroendocrine tumors (n=28) were stained with an anti-sortilin antibody. Immunoreactivity scores (the product of staining intensity (0, no staining; 1, weak staining; 2, moderate staining; 3, strong staining) and percentage of positive staining cells (0, no positive; 1, 0%-10% positive; 2, 11%-50% positive; 3, 51%-100% positive)) were compared between functional (f) and non-functional (nf) NETs. Representative image of a non-functional **(A)** and a functional **(B)** liver metastasis of a small intestinal NET. **(C-F)** Immunoreactivity in functional and non-functional **(C)** primary NETs (7 pancreatic NETs and 14 small intestinal NETs, 7 nf vs. 14 f) **(D)** well-differentiated small intestinal NETs (n=14, 4 nf vs. 10 f), **(E)** liver metastases of NETs (10 pancreatic, 1 lung, 15 small intestinal and 2 NETs of unknown origin, 12 nf vs. 16 f), and **(F)** liver metastases of well-differentiated small intestinal NETs (n=15, 4 nf vs. 11 f). **(G)** Sortilin expression analyzed by Western blot of 3 non-functional (nf) and 3 functional (f) liver metastases of well-differentiated small intestinal NETs. **p<0.01, ***p<0.001.

Data are presented as mean ± standard error of the mean (SEM). Statistical analysis was performed using Student’s t-test when comparing pairs of means, one-way ANOVA with Bonferoni’s Test when comparing more than two groups, and Spearman correlation for correlation analyses of ordinal data (*p < 0.05; ** p < 0.01; *** p < 0.001) with the GraphPad Prism 8 software (GraphPad Software, Inc., La Jolla, USA). Additionally, Welch’s correction was performed to compare the means of immunoreactivity in functional vs. non-functional NETs (**Figure 1**).

## Results

### Sortilin is a marker of functional NETs

First, we performed immunohistochemical analysis of a cohort of 49 well-differentiated NET samples for sortilin expression. This cohort was derived from 41 patients (22 male and 19 female, median age 58.4 years) and included 14 intestinal and 7 pancreatic primary tumors as well as 28 hepatic metastases of 15 intestinal, 10 pancreatic, 1 lung, and 2 unknown primary tumors. Of these, 30 tumors were functional and 19 non-functional. Staining intensity was estimated by an immunoreactivity score as previously described (10). We found high sortilin immunoreactivity in a subpopulation of NETs. Expression did not correlate with tumor site, sex, or Ki67 index (**Supplementary Figures 1A-E**). Interestingly, sortilin expression was twice as high in tumors that caused hormone-associated disease (**Figures 1A, B**). To exclude that the difference was due to bias between primary tumors and metastases, we performed a subgroup analysis of primary tumors only and confirmed about two times higher expression in primary tumors of patients with functional syndrome (**Figure 1C**). To further exclude bias due to the tissue origin of the NETs, we re-analyzed the subgroup of small intestinal tumors and again confirmed a twofold higher sortilin expression in functional NETs. (**Figure 1D**). The same result was observed for subgroup analysis of liver metastases of all NETs (**Figure 1E**) and of liver metastases from patients with small intestinal NETs only (**Figure 1F**). To confirm these findings, we performed Western blot analysis of six different liver metastases of small intestinal NETs and found higher sortilin expression in functional tumors (**Figure 1G**). Thus, our data indicate that high sortilin expression is a marker of functional NETs.

We also observed a moderate correlation between sortilin expression and patient age. This effect was attributed to the lower age of patients with a functional syndrome in our cohort, as no correlation with age was found when the analysis was restricted to functional tumor samples only (**Supplementary Figures 1F-H**).

### Sortilin inhibition decreases serotonin production in functional NET cell cultures

**Figure 2:**
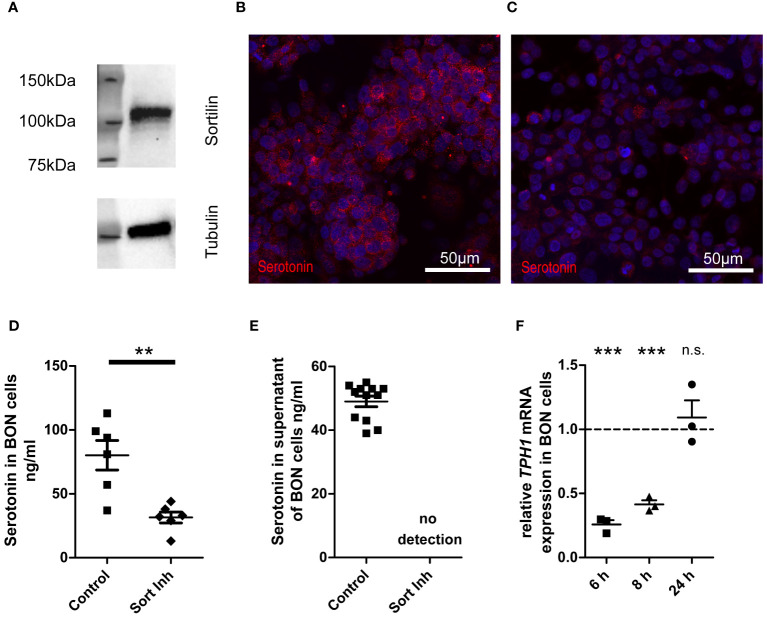
Impact of sortilin inhibition on serotonin content of NET cells. BON cells as a model of serotonin-expressing and -secreting NETs were treated for 24 h with the sortilin inhibitor AF38469 (10 µM). **(A)** Sortilin expression was analyzed by Western blot in BON cells (n=1). **(B, C)** BON cells stained for serotonin **(B)** without and **(C)** with 24 h sortilin inhibition. **(D)** Quantification of serotonin in BON cell lysates with and without 24 h sortilin inhibition (n=6 for each). **(E)** Quantification of serotonin in BON cell supernatants with and without 24 h sortilin inhibition (n=12 for each). **(F)** Relative TPH1 expression in relation to untreated BON cells 6, 8, and 24 h after addition of the sortilin inhibitor (n=3 for each). **p<0.01, ***p<0.001, n.s., not significant.

To examine if sortilin is directly involved in hormone production or secretion from NETs, we next analyzed the functional consequences of sortilin inhibition. For this, we focused on one of the most common functional syndromes, carcinoid syndrome, which occurs mainly due to the overproduction and secretion of serotonin (14). As a model system, we used the serotonin-secreting neuroendocrine BON cell line (15). Western blot analysis confirmed that these cells express sortilin (**Figure 2A**), as reported previously (10). Next, BON cells were cultured for 24 h in serotonin-free medium with or without addition (10 µM) of the small molecule sortilin inhibitor AF38469 (16). Immunofluorescence labeling for serotonin revealed that cells treated with the inhibitor contained less serotonin than untreated control cells (**Figure 2B**: control, **Figure 2C**: 24h sortilin inhibition). To quantify the results, we measured the serotonin content in cell lysates (**Figure 2D**) using high-performance liquid chromatography (HPLC). Compared to control cells, the cellular serotonin content was 60% lower in cells treated with sortilin inhibitor (80.17 ± 11.58 vs. 31.5 ± 4.28 ng/ml). Reduced intracellular serotonin concentration could be caused by reduced production or increased metabolism or secretion. To rule out the latter possibility, we quantified serotonin concentrations in the cell culture medium and could not detect any amount after sortilin inhibition (**Figure 2E**). Furthermore, we quantified 5-hydroxyindoleacetic acid (5-HIAA), the main metabolite of serotonin, in the supernatant and found a 67% decrease of 5-HIAA levels after sortilin inhibition indicating that an increased metabolism of serotonin is not the reason for reduced cellular serotonin concentrations (**Supplementary Figure 2**). To rule out that the reduced serotonin production resulted from any toxic effects of sortilin inhibition, we quantified the number of live cells by using the trypan blue assay but did not observe any effects on total cell number or the percent of live cells upon AF38469 treatment (**Supplementary Figures 3A, B**). Furthermore, inhibition of sortilin did not affect cell proliferation, as shown by the alamar blue proliferation assay (**Supplementary Figure 3C**).

To corroborate impaired serotonin production in cells treated with sortilin inhibitor, we performed qPCR for tryptophan hydroxylase 1 (TPH1), the key enzyme of serotonin synthesis. We observed a significant reduction of TPH1 expression by approximately 75% after 6 h and by approximately 60% after 8 h of sortilin inhibition compared to untreated cells (**Figure 2F**). This effect was restored after 24 h. In summary, sortilin inhibition leads to impaired serotonin production through decreased expression of TPH1.

### Sortilin inhibition decreases serotonin levels in differentiated enteroendocrine organoids

**Figure 3:**
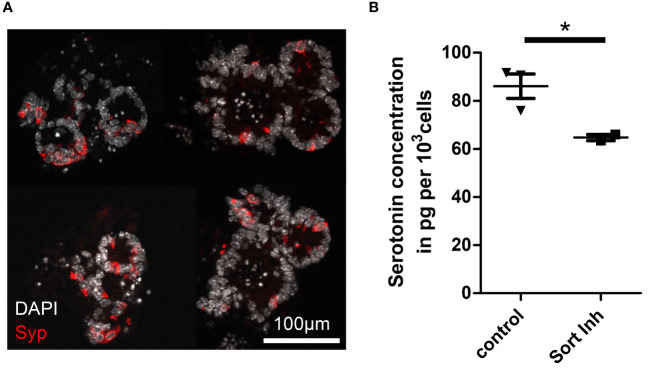
Impact of sortilin inhibition on serotonin content of enteroendocrine-differentiated organoids. **(A)** Fluorescence micrograph showing representative murine ileal organoids enriched for enteroendocrine cells (EECs). **(B)** Serotonin concentration per 1,000 cells in EEC-enriched organoids without (control) and with sortilin inhibition using AF38469 (10µM) (n=3 for control (2 biological replicates) and n=2 for sortilin inhibition (1 biological replicate), *p=0.048).

Although it is unknown why some NETs are functional and others non-functional, it is assumed that the basic mechanisms of hormone production and secretion in NETs are similar to those in healthy enteroendocrine cells (EECs), the suspected cell of origin of NETs (17–20). Therefore, we used murine intestinal organoids to assess the relevance of sortilin for hormone production and secretion from EECs (21). As the proportion of EECs in intestinal organoids is low, recapitulating the rare occurrence of EECs *in vivo* (0.1-1%), we applied growth factors to the culture medium to enrich organoids for EECs, as previously described (22, 23). Immunostaining for synaptophysin and qPCR for synaptophysin and chromogranin A confirmed enrichment of EECs (**Figure 3A**; **Supplementary Figures 4A, B**). EEC-enriched organoids produced sufficient amounts of serotonin to enable detection in pooled lysates (**Figure 3B**, control). Next, we treated EEC-enriched organoids with AF38469 for 4 days. After treatment, organoids showed a 25% lower concentration of serotonin per cell when compared to control conditions (**Figure 3B**, Sort Inh). This result indicates a role for sortilin in serotonin production in enteroendocrine cells. However, due to the small sample size of the treated group (n=2), additional validation of the mechanism should be performed in further studies.

## Discussion

In summary, we demonstrate that functional NETs express twice as high levels of sortilin than non-functional NETs, making it the first molecular marker of NET hormonal activity. Using cell culture and EEC-enriched organoids we demonstrate that sortilin inhibition leads to reduced serotonin levels. In BON cells, this was due to reduced serotonin synthesis, most likely through lower TPH1 expression. How sortilin inhibition leads to reduced TPH1 expression remains unclear at present but may entail direct or indirect molecular mechanisms, in line with the many activities of this multifunctional receptor (24–28). Another limitation of the current study is that we observed higher levels of sortilin expression also in functional pancreatic NETs. It is important to note that the functional syndrome of pancreatic NETs is generally not caused by serotonin. Further investigation is needed to identify the way of action in this condition.

In patients with carcinoid syndrome, the overproduction and secretion of hormones, including serotonin, leads to symptoms negatively influencing quality of life and, additionally, to a tumor-independent shortening in overall survival (4, 5, 29). Besides surgery, peptide radioreceptor therapy (PRRT) and local ablative therapies, there are only limited pharmaceutical treatment options available (29). These include somatostatin analogues, the TPH1 inhibitor Telotristat, which only has a strong effect in the treatment of diarrhea (30, 31), and interferon alpha, which has a low tolerability due to side effects (29). One reason for the limited pharmaceutical treatment options is our limited understanding of functional syndrome. Even the molecular reasons why some NETs are functional and some are not remain to be identified. Here, we report sortilin as a novel target for discriminating between functional and non-functional NETs. Furthermore, sortilin is not only a marker of functional NETs, it is also directly involved in the synthesis of one of the main hormones released by functional NETs: serotonin. As sortilin inhibition diminishes serotonin production, receptor antagonists may represent a novel therapeutic strategy for treating carcinoid syndrome. Sortilin is already an established drug target for other diseases. AL001, an anti-sortilin antibody, has currently reached a phase III study for treatment of frontotemporal dementia (NCT04374136), and TH1902, a drug consisting of docetaxel conjugated to a sortilin-targeting peptide, is currently being tested in a phase I study for treatment of several solid cancers (NCT04706962). These drug candidates could be repurposed to augment the landscape of pharmaceutical treatment options for functional NETs. However, it should be noted that the current study did not evaluate AL001 and TH1902.

In order to investigate the mechanisms underlying human diseases, models are indispensable. Especially for small intestinal NETs, which cause the majority of carcinoid syndromes, appropriate models other than cell lines are lacking (22). To our knowledge, there is only one animal model of small intestinal NETs (32). However, this model on RT2 background mice only showed tumor formation in 12 out of 30 mice and only 22% out of these tumors were serotonin-positive. Although their ability to cause functional syndrome has not yet been assessed, the small proportion of serotonin-positive tumors raises doubts over the suitability of this model. Recently, attempts were made to use patient-derived organoids as a model of small intestinal NETs (33, 34), but have so far failed to model functional NETs. To our knowledge, the only functional NET organoid model described was developed by Kawasaki et al. (33), but consists of a gastrin-producing organoid line from a gallbladder NET and thus does not model carcinoid syndrome. Here, we used EEC-enriched normal intestinal organoids to explore hormone production and secretion. These organoids do indeed seem to be suitable as a surrogate model for functional small intestinal NETs, as EECs are widely accepted as the cell of origin of NETs and the mechanisms of hormone production do not differ between EECs and NETs (17–20). Our finding that sortilin inhibition causes decreased serotonin levels in these organoids underpins its role in carcinoid syndrome of functional NETs and makes it a novel potential target for treating this syndrome.

## Data availability statement

The original contributions presented in the study are included in the article/**Supplementary Material**. Further inquiries can be directed to the corresponding authors.

## Ethics statement

The studies involving humans were approved by Ethics Committee of the Charité - Universitätsmedizin Berlin (No EA1/229/17). The studies were conducted in accordance with the local legislation and institutional requirements. The participants provided their written informed consent to participate in this study. The animal study was approved by Landesamt für Gesundheit und Soziales, Berlin, T-CH0032/20. The study was conducted in accordance with the local legislation and institutional requirements.

## Author contributions

FB: Conceptualization, Data curation, Formal analysis, Investigation, Writing – original draft, Writing – review & editing. AW: Investigation, Writing – review & editing. YG: Investigation, Writing – review & editing. IE: Investigation, Writing – review & editing. NA: Investigation, Writing – review & editing, Resources. MB: Formal analysis, Resources, Writing – review & editing. TW: Conceptualization, Resources, Writing – review & editing, Formal analysis. BW: Conceptualization, Funding acquisition, Supervision, Writing – review & editing. MS: Conceptualization, Funding acquisition, Project administration, Resources, Supervision, Writing – review & editing.
