# Supplementary material for: Elevated sortilin expression discriminates functional from non-functional neuroendocrine tumors and enables therapeutic targeting
Source: Front Endocrinol (Lausanne). 2024 Apr 17;15:1331231. doi: 10.3389/fendo.2024.1331231 (PMC11061435; doi:10.3389/fendo.2024.1331231)
Supplement: Supplementary file 1 [file DataSheet_1.docx]

Supplementary Material

**Supplementary figure 1**

**
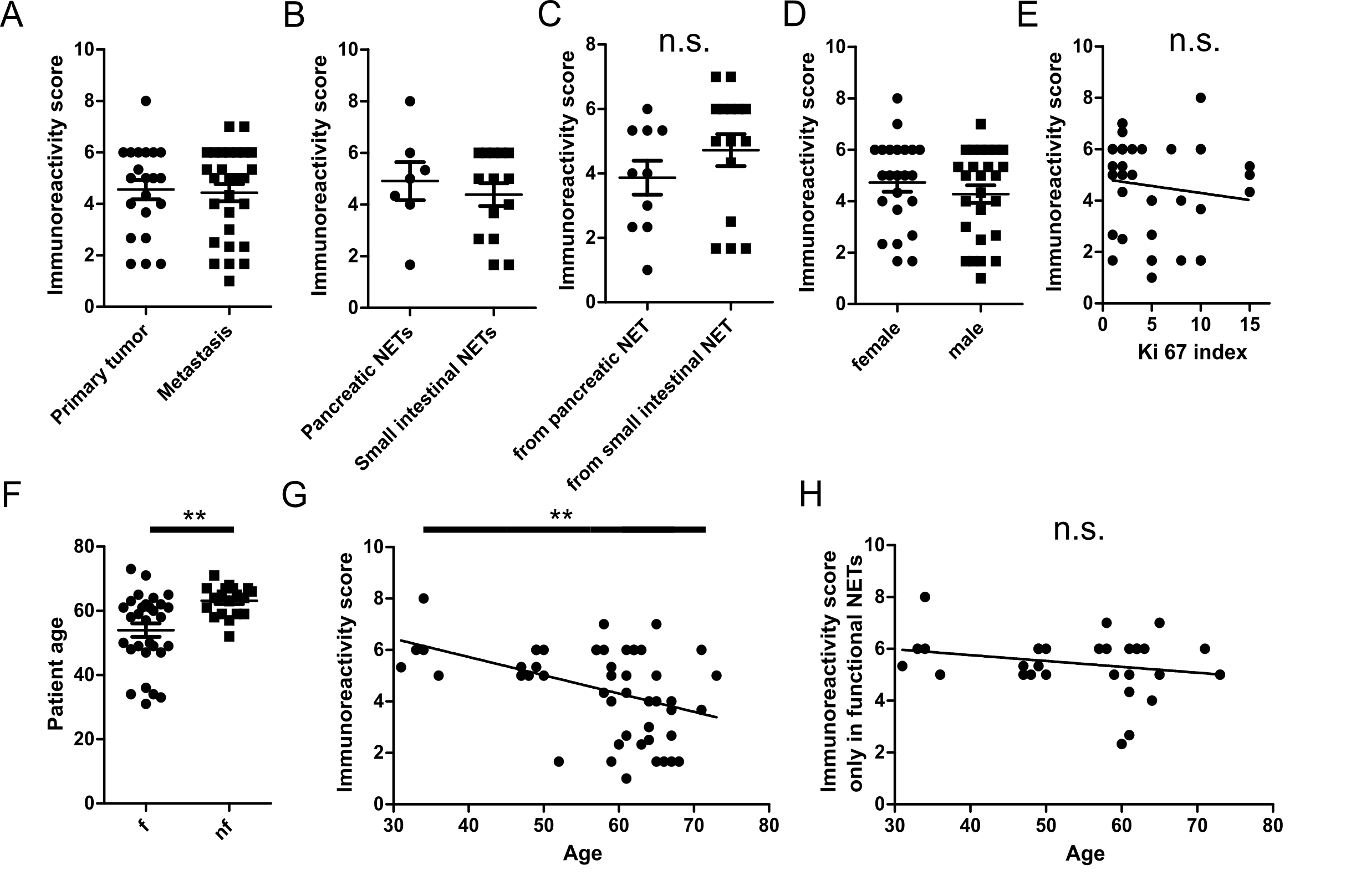
**

**Supplementary figure 1 Sortilin expression in correlation to the tumor site, sex, Ki67 index, and patient age.** Immunoreactivity scores for sortilin in dependence of primary tumor or metastasis (A), pancreatic or small intestinal primary tumor (B), origin of liver metastasis (C), sex (D), or Ki67 index (E). (F) Patient age in dependence of functionality. Due to the significantly lower age of patients with functional syndrome in our cohort, the sortilin immunoreactivity is negatively correlated with patient age (G, Spearman r= -0.4485, p=0.0012). When analyzing only functional tumors, this correlation no longer exists (H). f: functional. nf: non-functional. n.s.: not significant, **p<0.01.

**Supplementary figure 2**

**
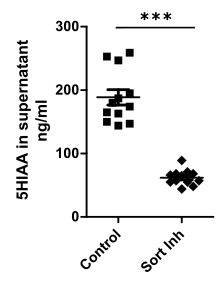
**

**Supplementary figure 2 Impact of sortilin inhibition on 5-hydroxyindoleacetic acid (5-HIAA) concentration in cell culture supernatant.** BON cells were cultured in control conditions or treated for 24 h with the sortilin inhibitor AF38469. Afterwards the concentration of the main serotonin metabolite 5-HIAA was estimated in the supernatant by HPLC (n=12 for each). *** p<0.001.

**Supplementary figure 3**


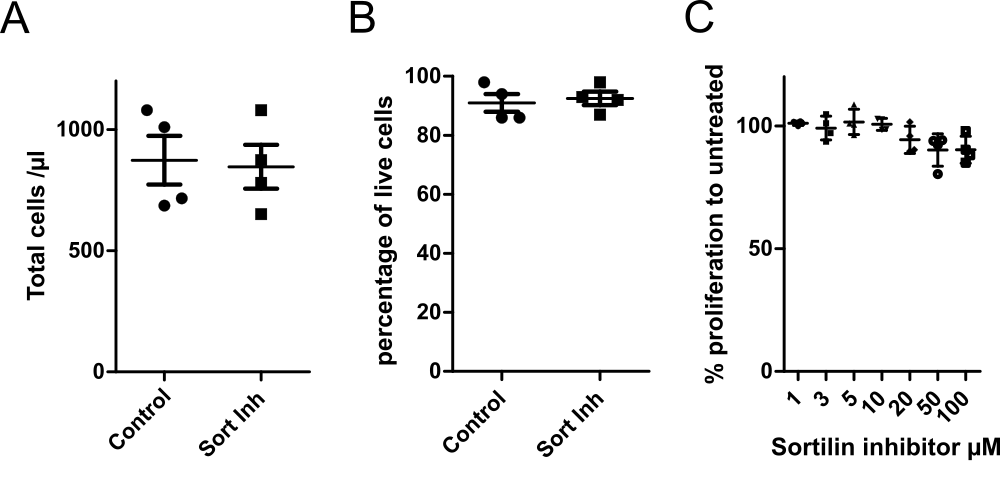


**Supplementary figure 3 Sortilin inhibition has no impact on BON cell number or proliferation.** BON cells were cultured in control conditions or treated for 24 h with the sortilin inhibitor AF38469. (A) Total cell number and (B) total number of live cells. (C) Proliferation of BON cells treated with different concentrations of AF38469 compared to untreated cells. n=4 for each experiment and group.

**Supplementary figure 4**

**
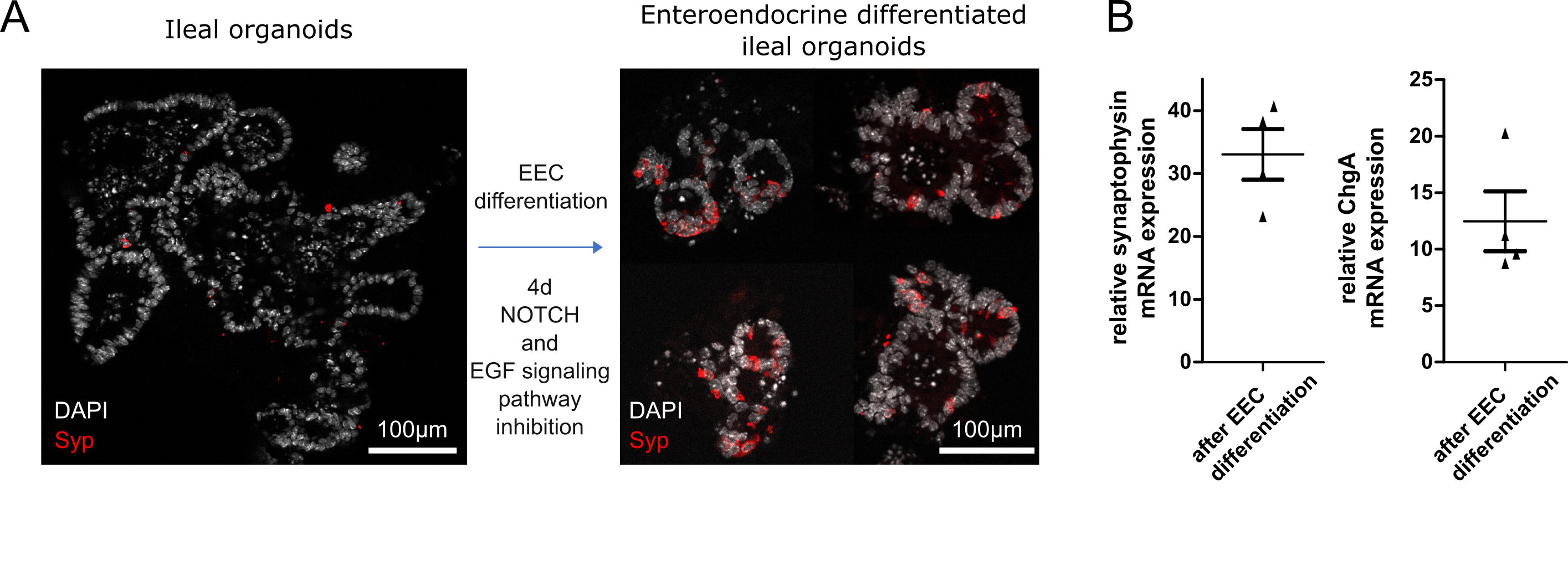
**

**Supplementary figure 4 Generation of enteroendocrine-differentiated organoids.** Murine ileal organoids contain only a very limited number (0.1-1%) of enteroendocrine cells (EECs), which were defined by expression of the marker protein synaptophysin (A, left image). 3 days after passaging, ileal organoids were treated for 4 days with DAPT (10 µM) to inhibit the NOTCH signaling pathway and with PD0325901 (1µM) to inhibit the EGF signaling pathway by MEK inhibition as previously described (23). After treatment, organoids were drastically enriched with EECs (A, right image). (B) qPCR demonstrates a strong increase in transcription of the EEC marker genes synaptophysin and chromogranin A (ChgA).
